# Immune Checkpoint Inhibitors: A Promising Choice for Endometrial Cancer Patients?

**DOI:** 10.3390/jcm9061721

**Published:** 2020-06-03

**Authors:** Lucia Musacchio, Serena Maria Boccia, Giuseppe Caruso, Giusi Santangelo, Margherita Fischetti, Federica Tomao, Giorgia Perniola, Innocenza Palaia, Ludovico Muzii, Sandro Pignata, Pierluigi Benedetti Panici, Violante Di Donato

**Affiliations:** 1 Department of Maternal and Child Health and Urological Sciences, University of Rome “Sapienza”, Policlinico “Umberto I”, 00161 Rome, Italy; lucia.musacchio@uniroma1.it (L.M.); serenamaria.boccia@uniroma1.it (S.M.B.); giusi.santangelo@uniroma1.it (G.S.); margherita.fischetti@uniroma1.it (M.F.); federica.tomao@uniroma1.it (F.T.); giorgia.perniola@uniroma1.it (G.P.); innocenza.palaia@uniroma1.it (I.P.); ludovico.muzii@uniroma1.it (L.M.); pierluigi.benedettipanici@uniroma1.it (P.B.P.); violante.didonato@uniroma1.it (V.D.D.); 2 Department of Urology and Gynecology, Istituto Nazionale Tumori IRCCS “Fondazione G. Pascale”, 80131 Naples, Italy; s.pignata@istitutotumori.na.it

**Keywords:** endometrial cancer, immune checkpoint inhibitors, targeted therapy

## Abstract

Although around 80% of endometrial cancers are diagnosed at early stages and present with a 5-year survival rate exceeding 95%, patients with advanced and recurrent disease show a poor prognosis and low response rates to standard chemotherapy. In the era of targeted therapy, the great advances in the understanding of programmed death-ligand 1 (PD-L1) upregulation in cancer cells, which is responsible for tumor immune escape, have contributed to the increasing interest in immune checkpoint inhibitors as a promising strategy for the treatment of several refractory solid malignancies, including endometrial cancer. Several clinical trials have investigated the efficacy and safety of immune checkpoint inhibitors in endometrial cancer, which already led to the approval of the anti-programmed cell death protein 1 (anti-PD-1) antibody pembrolizumab as a satisfactory alternative for selected patients with unresectable or metastatic disease. As the future of cancer treatment will probably rely on combination therapy strategies, currently, innovative ongoing trials are exploring the potential role of immune checkpoint inhibitors associated with chemotherapy, radiotherapy, and other targeted therapies. Moreover, further research is warranted to discover new specific biomarkers that can accurately predict the response to immunotherapy.

## 1. Introduction

Endometrial cancer (EC) is the most common gynecological cancer in developed countries, with a lifetime risk of 2.8% [1]. The American Cancer Society estimates that, in 2020, about 65,620 new cases will be diagnosed in the United States and around 12,590 women will die from uterine cancers [2]. Around 80% of EC are diagnosed when the disease is limited to the uterus, with a five-year survival rate of over 95% [3]. However, the prognosis is significantly worse in the cases of regional spread or distant disease (68% and 17%, respectively) [4]. According to international guidelines, treatment for patients with early stage EC is based on surgery alone or in combination with pelvic radiation therapy, whereas patients with advanced disease are candidates for systemic chemotherapy, with carboplatin-paclitaxel doublet representing the most effective scheme [5]. Patients with metastatic or recurrent disease have a poor prognosis as they exhibit low response rates to cytotoxic chemotherapy; thus, it is necessary to explore new therapeutic approaches in order to provide them with the best care. In this review, we discuss the scientific rationale behind the use of immune checkpoint inhibitors in advanced and recurrent EC, exploring both published and ongoing clinical trials. 

## 2. Background

The human immune system consists of two main complementary subsystems: (1) the innate immune system, which gathers dendritic cells, natural killer (NK) cells, macrophages, neutrophils, eosinophils, basophils, and mast cells and does not depend on a prior stimulation by antigens [6], and (2) the adaptive immune system, which includes B lymphocytes, cluster of differentiation 4 (CD4+) helper T lymphocytes, and cluster of differentiation 8 (CD8+) cytotoxic T lymphocytes and requires a prior antigen exposure mediated by antigen-presenting cells (APCs) for its activation [7]. Immune cells can recognize and eliminate cancer cells through the identification of tumor-specific antigens (TSA) and tumor-associated antigens (TAA) [6]. Specifically, T cells can recognize TAA and TSA peptide small fragments displayed on the surface of antigen-presenting cells (APCs) after being loaded on major histocompatibility complex (MHC) class I and II molecules [8]. Physiologically, the immune system needs to be finely regulated in order to act against pathogens without affecting self-tissues (self-tolerance). Generally, the immune response activation is elicited if two positive signals are present: the first one arises from the interaction between MHC molecules and T-cell receptors (TCR), while the second one imposes that the co-stimulatory receptor cluster of differentiation 28 (CD28), expressed on T cells surface, binds its ligand B7 on APCs [9]. In order to avoid an autoimmune reaction, there are two main inhibitory pathways: the first one depends on the cytotoxic T lymphocyte antigen-4 (CTLA-4), which acts as a competitive inhibitor for the ligand B7 and is mostly expressed within secondary lymphoid organs [10]; the second one is related to the interaction between PD-1 receptor on T cells and PD-L1/2 on the tumor cells surface, and it occurs more frequently within peripheral tumor microenvironment (TME) [11,12]. The PD-1 pathway is an immune checkpoint involved in the regulation of T cell differentiation and apoptosis [13]. PD-1 is a protein receptor expressed by multiple immune cells, including T cells, B cells, NK cells, dendritic cells, and monocytes [14]. The interaction between PD-1 and its ligands, PD-L1 and PD-L2, inhibits cytotoxic T cell activation and differentiation [15]. Particularly, PD-L1 has been shown to be the most important ligand and is upregulated in cancer cells in order to escape the immune surveillance [16]. Indeed, antibodies targeting PD-1 and PD-L1 can block tumor-generated immunosuppression, thus preserving the immune response against cancer [17]. In light of these observations, immune checkpoint inhibitors appeared as a promising choice for the treatment of different solid and refractory malignancies, and in recent years, several studies have been conducted to assess the potential role of specific biomarkers in predicting the response to immunotherapy.

Historically, EC has been classified according to Bokhman’s model into two subtypes, based on clinicopathological and molecular features. The type I EC is the most common (60–70% of cases) and includes grades 1–2 endometrioid histology. It is associated with a high expression of estrogen receptors (ERs) and progesterone receptors (PgRs) and a good prognosis (median 5-year survival rates of 85.6%). On the other hand, the type II represents about 30–40% of all EC, typically embracing serous, clear cell or grade 3 endometrioid histology, and is estrogen-independent [18]. It is typically more aggressive than type I and is associated with a poor prognosis, even when diagnosed at early stages [19,20].

In 2013, the Cancer Genome Atlas Research Network, given the new advances on the understanding of EC genomic landscape, introduced a new molecular classification that could change adjuvant treatment for women with an aggressive disease, based on four categories: polymerase ε (POLE)-ultramutated, microsatellite instability-high (MSI-H), copy-number low, and copy-number high [21]. Each of these molecular subclasses present a distinct profile of tumor aggressiveness and thus a different prognosis. The multicenter, randomized phase III trial PORTEC-4a (NCT03469674) is investigating the impact of molecular risk factors in assigning adjuvant treatment for women with stages I–II high-intermediate risk endometrial cancer. POLE-ultramutated and MSI/Mismatch Repair Deficiency (MMRd) tumors are associated with a better prognosis compared to the other subgroups. Specifically, POLE-ultramutated tumors show a better progression free survival (PFS) when compared to other tumors with the same histotype, grade, and stage [22,23,24]. Several authors have reported that MSI-H/MMRd colorectal cancers are significantly associated with a better prognosis following conventional chemotherapy [25,26] and immunotherapy treatment [27]. Indeed, POLE-ultramutated and MSI-H molecular subclasses are characterized by high CD3+/CD8+ tumor-infiltrating lymphocytes and an overexpression of PD-1 and PD-L1 [28,29,30,31,32]. Specifically, mutations that occur in the domain of the POLE gene cause a specific phenotype characterized by a high mutational load, with increased neoantigen expression and an important activation of the patient’s immune system [33,34]. Similarly, MSI-H/MMRd cancers are characterized by extremely high numbers of somatic mutations, and several studies have evaluated the relationship between MMR status and the expression of PD-1 and PD-L1 in solid tumors including EC [35,36]. Interestingly, Yamashita et al. reported higher cytotoxic T cells (CD8+) infiltration and PD-1/PD-L1 expression in the MMR-deficient (MMRd) group compared to the MMR-proficient (MMRp) among 149 endometrial cancer patients [37]. The high immunogenicity of these tumors explains the strong rationale behind the use of immunotherapy in these subgroups of cancers. In light of these observations, EC can represent an ideal target for immune checkpoint inhibitors and the use of MMRd/MSI status could be a predictor of the response to PD-1 blockade in endometrial cancer.

## 3. Clinical Evidence

Several clinical trials have been published regarding the role of immune checkpoint inhibitors in endometrial cancer (Table 1).

### 3.1. Anti-PD-1: Pembrolizumab

In 2015, Le et al. first evaluated the clinical activity of pembrolizumab, an anti-PD-1 immune checkpoint inhibitor, in 41 patients presenting with progressive metastatic colorectal cancer (CRC) with or without MMR deficiency or other MMRd cancers. Pembrolizumab was administered intravenously (IV) at a dose of 10 mg/kg every 14 days in three different cohorts of patients, which included, respectively, MMRd CRC, MMRp CRC, and MMRd noncolorectal cancers, among which there were also 2 cases of EC. Co-primary endpoints were the immune-related objective response rate (ORR) and the 20-week immune-related progression-free survival (PFS). In the MMRd CRC group, the estimated ORR and the 20-week PFS were 40% and 78%, respectively, versus 0% and 11% reported for MMRp CRC patients. In the MMRd cohort including two EC patients, the ORR and PFS were 71% and 67%, respectively [38].

In 2016, Mehnert et al. published the case of a single patient with a rapid and persistent (more than 14 months) clinical response to anti-PD-1 pembrolizumab treatment. The molecular analysis of a pretreatment biopsy revealed a POLE-ultramutated EC, thus suggesting that the identification of a POLE mutation might have a predictive value of treatment response to pembrolizumab. However, these data need to be further investigated to be confirmed [39]. 

In 2017, Ott et al. published the results of the phase Ib KEYNOTE-028 trial, which enrolled 24 advanced EC patients treated with pembrolizumab 10 mg/kg IV administered every 2 weeks for a maximum of 24 months or until confirmed disease progression, intolerable toxicity, or death. The ORR reported was 13%. Three women showed a partial response, and among them, one had POLE mutations, whereas three patients achieved a stable disease. Six-month PFS and OS were 19.0% and 68.8%, respectively. Thirteen patients (54.2%) experienced treatment-related adverse events (TRAEs), such as fatigue (20.8%), pruritus (16.7%), pyrexia (12.5%), and decreased appetite (12.5%). Grade 3 adverse events (AEs) were reported in 4 patients, including asthenia, back pain, anemia, hyperglycemia, hyponatremia, pyrexia, and diarrhea. No patient experienced grade 4 AEs or any grade immune-mediated AEs, discontinued treatment because of an AE, or experienced treatment-related death. [40].

In a recent phase II trial, Makker et al. tested the anti-PD-1 pembrolizumab at a dosage of 200 mg IV every 3 weeks in combination with lenvatinib, a multikinase-inhibitor of vascular endothelial growth factor (VEGFR), at a dosage of 20 mg daily orally in 108 advanced recurrent EC patients. The primary endpoint was the ORR at 24 weeks, according to the response evaluation criteria in solid tumors (RECIST) criteria 1.1. Forty-two (38.0%) patients had an objective response to lenvatinib plus pembrolizumab. The median PFS and overall survival (OS) were 7.4 months and 16.7 months, respectively. For patients with MSS/MMRp tumors, the ORR was 37.2%, whereas for patients with MSI-H/MMRd tumors, the ORR was 63.6%. The median duration of response (DOR) was not estimable (NE) (95% CI, 7.4 months to NE) for patients with MSS/MMRp tumors and was 21.2 months (95% CI, 7.3 months to NE) for patients with MSI-H/MMRd tumors. The most frequently reported any-grade AEs were hypertension (66 (61%)), fatigue (56 (51.9%)), diarrhea (57 (52.8%)), and hypothyroidism (48 (44.4%)). Grade 3–4 AEs occurred in 75 (69.4%) patients [41].

Results from the Keynote-158 study demonstrated the clinical benefit of pembrolizumab for previously treated unresectable or metastatic MSI-H/MMRd noncolorectal cancer patients. Among 233 patients enrolled, 47 were EC patients. Eligible participants received pembrolizumab 200 mg IV every 21 days for 35 cycles or until documented disease progression, unacceptable toxicity, or patient/investigator decision. The primary endpoint was ORR, defined as the proportion of patients with confirmed complete/partial response per RECIST 1.1. An ORR of 57.1% was reported for EC patients, with a median PFS of 25.7 months. One hundred and fifty-one of 233 patients (64.8%) experienced TRAEs, and among them, 34 (14.6%) had grade 3–5 AEs. The most common toxicities were fatigue (14.6%), pruritus (12.9%), diarrhea (12.0%), and asthenia (10.7%;). Most frequent grade 3 AEs were increased gamma-glutamyltransferase (1.7%) and pneumonitis (1.3%). Three patients had grade 4 AEs: one patient had Guillain–Barre syndrome (a patient with gastric cancer), one had increased alanine aminotransferase (ALT), and another one had decreased neutrophil count and enterocolitis [42].

### 3.2. Anti-PD-1: Nivolumab

In 2016, Santin et al. reported two cases of recurrent/metastatic EC, respectively a mixed clear cell and endometrioid (CC/EAC) POLE-ultramutated EC and a serous MSH6-mutated EC, not responding to surgery, chemotherapy, and radiotherapy and treated with nivolumab, a novel anti-PD-1 antibody, administered IV at a dosage of 3 mg/kg once biweekly. Both tumor samples evaluated in the pretreatment setting showed moderate peri- and intratumoral lymphocytic infiltrate (especially CD8 T cells) and moderate PD-1 and PD-L1 expression. Patients showed a persistent clinical response to nivolumab without severe toxicities, as confirmed by a computed tomography (CT) scan performed, respectively, at 7 and 9 months from the beginning of treatment [43].

### 3.3. Anti-PD-1: Dostarlimab

Preliminary data from the GARNET study were presented at the 2019 Society for Gynecologic Oncology (SGO) annual meeting on women’s cancer. One hundred and ten EC patients received dostarlimab 500 mg every 3 weeks. The ORR was 27.7% (50% in MSI-H and 19.1% in MSS). Sixty-eight EC patients (68%) experimented at least one TRAE. Grade ≥ 3 AEs were reported in 13 patients (11.8%), and the most common was increased serum levels of aspartate aminotransferase [44].

### 3.4. Anti-PD-L1: Atezolizumab

Fleming et al. presented the results of a phase Ia clinical trial in which atezolizumab, an anti-PD-L1 antibody, was tested in 15 women with advanced and recurrent EC with microsatellite stability (MSS), MSI-H, or microsatellite unknown status. Patients received atezolizumab 1200 mg or 15 mg/mq IV in monotherapy every 21 days. Two women showed a partial response, while another two achieved a stable disease with an ORR of 13% and a disease control rate (DCR) of 27%. Both responders had a PD-L1 expression above 5% and presented, respectively, MSS and MSI-H status. It was reported a median PFS of 1.7 months and a median OS of 9.6 months. Only two patients experienced severe drug-related AEs (colitis and rash), whereas no G4–5 adverse events were recorded [45].

### 3.5. Anti-PD-L1: Avelumab

Konstantinopoulos et al. published the results of a phase II trial, in which avelumab was tested in patients with MMRd/MMRp-recurrent or -persistent endometrial cancer. Thirty-three patients were enrolled. Avelumab was administered at 10 mg/kg IV every 2 weeks until progression or unacceptable toxicity. No patient with POLE-ultramutated tumor was enrolled in MMRd and MMRp cohorts. Co-primary endpoints were PFS at 6 months (PFS6) and ORR according to RECIST criteria 1.1. The MMRp cohort was closed at the first stage because only one out of 16 patients exhibited both ORR and PFS6 responses. The MMRd cohort achieved the primary endpoint of four objective responses after the accrual of only 17 patients. Of 15 patients who received avelumab, four exhibited an objective response (1 complete response and 3 partial responses; ORR, 26.7%; 95% CI, 7.8% to 55.1%) and 6 (including all four objective responses) PFS6 responses (PFS6, 40.0%; 95% CI, 16.3% to 66.7%). Responses were observed in the absence of PD-L1 expression. Twenty-two patients experienced TRAEs. Particularly, 6 patients reported grade 3 toxicity, including anemia, sinus bradycardia, hypothyroidism, diarrhea, myositis, and rash acneiform [46].

### 3.6. Anti-PD-L1: Durvalumab

Data from the PHAEDRA study was presented at the American Society of Clinical Oncology (ASCO) 2019 and showed the activity of durvalumab as a single agent in MMRd and MMRp cohorts. Seventy-one patients with advanced EC were enrolled: 35 patients with MMRp progressing after 1–3 lines of chemotherapy and 36 patients with MMRd progressing after 0–3 lines of chemotherapy. All patients received durvalumab at dosage of 1500 mg IV every 4 weeks. Among MMRd patients, the ORR was 40% (4 complete responses and 10 partial responses), whereas within the MMRp group, the ORR was 3% (one partial response). Adverse events occurred in 14 patients, including hyperthyroidism, hypothyroidism, pneumonitis, and hepatitis [47].

## 4. Main Ongoing Trials

Immune checkpoint inhibitors are under evaluation in several phase I, II, and III clinical trials, alone or in combination with other drugs (Table 2). NCT03572478 is a phase Ib/IIa study on the combination of nivolumab with the poly (ADP-ribose) polymerase (PARP) inhibitor rucaparib in metastatic or recurrent EC patients. In phase Ib, patients will receive rucaparib plus nivolumab in 4-week cycles, while in phase IIa, patients will be randomized to receive nivolumab alone, rucaparib alone, or rucaparib in combination with nivolumab. The primary endpoint is to evaluate the dose-limiting toxicities (DLT) rate for the combination of rucaparib and nivolumab [48]. Nivolumab is also being studied in a phase II clinical trial (NCT02982486) in association with ipilimumab in patients with nonresectable/metastatic MMRd EC. Patients will receive nivolumab 240 mg IV every 2 weeks plus ipilimumab 1 mg/mq IV every 6 weeks. The primary endpoint is ORR according to RECIST 1.1 [49]. NCT03241745 is a single-center, non-randomized, open-label, phase II study with the aim to assess the safety of nivolumab in patients with MSI/MMRd hypermutated uterine cancers, including endometrial carcinoma. Patients will receive nivolumab 480 mg IV once every 4 weeks until disease progression or unacceptable toxicity. The primary endpoint is PFS [50].

Moreover, pembrolizumab is being investigated in phase I, II, and III clinical trials. NCT02549209 is a single-arm, open-label, multi-center phase II study in patients with newly diagnosed stage III/IV or recurrent EC that aims to assess the association of pembrolizumab with carboplatin/paclitaxel therapy. Patients with no prior therapy will receive pembrolizumab 200 mg plus carboplatin AUC 6 and paclitaxel 175 mg/mq every 21 days, whereas those with prior external beam radiation therapy (EBRT) and/or platinum-based chemotherapy must initiate paclitaxel and carboplatin at a reduced dose (paclitaxel 135 mg/mq plus carboplatin AUC 5). The primary endpoint is the ORR [51].

The TOPIC study (NCT03276013) is non-randomized, single-arm, multi-center, phase II study of pembrolizumab in combination with doxorubicin in patients with recurrent/metastatic EC. Patients will receive doxorubicin 60 mg/kg IV every 3 weeks for up to 9 cycles in combination with pembrolizumab (MK-3475) 200 mg IV every 3 weeks. The primary endpoint is the PFS6 [52]. NCT03517449 (KEYNOTE-775) is a multi-center, open-label, randomized, phase III trial investigating the efficacy and safety of combined lenvatinib and pembrolizumab compared to treatment with doxorubicin or paclitaxel in patients with advanced EC. Women in the experimental arm will receive pembrolizumab 200 mg IV on day 1 of each 21-day cycle plus lenvatinib 20 mg orally once daily during each 21-day cycle for up to 35 cycles. Patients in the comparator arm will receive doxorubicin 60 mg/mq IV on day 1 of each 21-day cycle for up to a maximum cumulative dose of 500 mg/mq or paclitaxel 80 mg/mq IV on a 28-day cycle (3 weeks of paclitaxel once a week and 1 week without paclitaxel). Co-primary endpoints are PFS and OS [53].

Two phase III clinical trials are evaluating the efficacy of pembrolizumab combined with other drugs for advanced or recurrent EC patients. In the randomized, placebo-controlled NCT03914612 study, patients will receive pembrolizumab every 21 days in association with carboplatin and paclitaxel for up to 6 cycles and then pembrolizumab every 3 weeks for up to 29 cycles as maintenance in the absence of disease progression or unacceptable toxicity [54]. The randomized, open-label NCT03884101 trial aims to compare the efficacy of pembrolizumab 200 mg IV on day 1 of each 21-day cycle in association with lenvatinib 20 mg orally once daily versus standard chemotherapy [55]. In both clinical trials, the primary endpoint is PFS.

MITO END-3 (NCT03503786) is a randomized, phase II clinical trial which compares carboplatin/paclitaxel to carboplatin/paclitaxel plus avelumab in advanced or recurrent EC. Patients in the experimental arm will receive carboplatin AUC 5, paclitaxel 175 mg/mq, and avelumab 10 mg/kg every 21 days for 6–8 cycles plus avelumab 10 mg/kg as maintenance every 14 days until disease progression or unacceptable toxicity. The primary endpoint is PFS [56].

Furthermore, durvalumab is currently under evaluation in several clinical trials. NCT04269200 is a randomized, multicenter, double-blind, placebo-controlled, phase III study which aims to assess the efficacy and safety of durvalumab in combination with platinum-based chemotherapy (paclitaxel plus carboplatin) followed by maintenance durvalumab with or without olaparib for patients with newly diagnosed advanced or recurrent EC. In cohort A, patients will receive carboplatin/paclitaxel plus durvalumab placebo followed by maintenance durvalumab placebo and olaparib placebo; in cohort B, patients will undergo platinum-based chemotherapy and durvalumab followed by maintenance durvalumab and olaparib placebo; and in cohort C, they will receive platinum-based chemotherapy and durvalumab followed by maintenance durvalumab and olaparib. PFS is the primary endpoint [57].

Finally, the EndoBARR trial (NCT03694262) is an open label, non-randomized multicenter, phase II trial evaluating the efficacy and safety of combined rucaparib, atezolizumab, and bevacizumab in recurrent and progressive EC. Atezolizumab will be administered at dosage of 1200 mg IV (every 3 weeks) together with bevacizumab 15 mg/mq (every 3 weeks) and rucaparib 600 mg twice daily. The primary endpoint is ORR [58].

## 5. Toxicities

Understanding the spectrum of the unique toxicities associated with immune checkpoint inhibition is crucial, as they may be acute and life-threatening. The precise pathophysiology underlying immune-related AEs is unknown, although it appears related to the role that immune checkpoints play in maintaining immunologic homeostasis. As CTLA-4 and PD-1 agents act differently, their use causes different immune-related AEs. Generally, the PD-1 blockade seems to be better tolerated and has a lower AE frequency than the CTLA-4 blockade. Moreover, CTLA-4-related AEs increase with increasing dose whereas PD-1-related AEs seem not to be dose-related [59]. Overall, AEs affect over two-thirds of patients. Severe immune-related AEs occur in 10% to 20% of cases treated with CTLA-4 inhibitors, whereas their frequency is lower (1% to 2%) in patients treated with PD-1 inhibitors [60]. Immune-related AEs usually develop within the first few weeks to months after treatment initiation, and there is currently no known predisposing risk factor. Furthermore, most studies indicate that prolonged treatment does not result in an increased incidence of immune-related AEs [61]. A current area of investigation is whether the degree of side effects is correlated with treatment response, though durable responses have been seen in the absence of severe reactions. Although any organ system can be affected, immune-related AEs most commonly involve gastrointestinal tract, endocrine glands, skin, and liver. Less often, the central nervous system and cardiovascular, pulmonary, musculoskeletal, and hematologic systems are involved [62]. The management of all potential immune-related adverse events requires a multidisciplinary approach.

## 6. Conclusions and Future Perspectives

Over the last years, immunotherapy has meaningfully changed the paradigm of care of several solid tumors and, currently, it represents an active area of research aiming to identify which types of cancer could benefit most from it. Recently, the great advances in the understanding of cancer genome have contributed to the increasing interest in immune checkpoint inhibitors as a promising alternative strategy for refractory cancers [63,64]. Based on the results from several clinical trials, in 2017, the Food and Drug Administration (FDA) approved pembrolizumab for patients with unresectable or metastatic MSI-H or MMRd solid tumors, including endometrial cancer. Moreover, data coming from Keynote 146 have led to the approval of the combination of pembrolizumab with lenvatinib in patients with highly pretreated, advanced non-MSI-H, or MMRd endometrial carcinoma, not amenable to surgery or radiation. Results from several clinical trials have shown that MMRd EC patients respond better to immune checkpoint inhibitors than MMR-p. However, there was no stratification of MSI-H EC tumors according to their sporadic or inherited Lynch syndrome (LS) origins. Since MSI-H tumors display an increased immunogenicity compared with MSS tumors, some authors have investigated differences in immune cell populations between sporadic and LS MSI-H endometrial cancer, showing that these two groups are distinct immunological entities with different types of immune cells in stroma and tumor, respectively [65,66]. In conclusion, given the evident benefit found in MSI-H/MMRd cancer patients treated with immune checkpoint inhibitors, all EC patients should be tested to define the MMR status at the time of diagnosis, including the immune-infiltrate and the expression of PD-1 and PD-L1, in order to establish their future eligibility for immunotherapy treatment [67,68]. Indeed, as the future of cancer treatment will rely on combination therapy strategies, several ongoing studies are evaluating the efficacy of immune checkpoint inhibitors associated with other immunotherapeutic agents, chemotherapy, radiotherapy, and targeted therapies in endometrial cancer. Interestingly, clinical evidence has suggested a strong correlation between PARP inhibition and PD-1/PD-L1 upregulation in different types of cancer. Therefore, the combined use of PARP inhibitors and anti-PD-L1 agents could show synergistic effects [69]. Further research is warranted to find new combination therapy strategies and to discover new specific biomarkers that can accurately predict the response to immunotherapy.

## Figures and Tables

**Table 1:** Clinical evidence on immune checkpoint inhibitors in endometrial cancer.

Authors, Years	Phase	Patient Population	Treatment	Findings
Le et al., 2015	II	41 pts with metastatic cancer with or without MMRd, including EC	Pembrolizumab 10 mg/kg IV every 14 days	**MMRd CRC** ORR: 40% PFS12: 78% **MMRp CRC** ORR: 0% PFS12: 11% **MMRd non-CRC** ORR: 71% PFS12: 67%
Ott et al., 2017	Ib	24 pts with PD-L1 positive locally advanced or metastatic EC	Pembrolizumab 10 mg/kg IV every 2 weeks up to 24 months	ORR: 13% PFS: 1.8 months
Fleming et al., 2017	Ia	15 pts with advanced or recurrent EC	Atezolizumab 1200 mg or 15 mg/kg IV every 3 weeks	ORR: 13% PFS: 1.7 months OS: 9.6 months
Makker et al., 2019	II	108 pts with advanced or recurrent EC	Pembrolizumab 200 mg IV every 3 weeks plus Lenvatinib 20 mg orally daily	ORR (24 weeks): 38.0% DOR: 21.2 months PFS: 7.4 months OS: 16.7 months
Kostantinopoulos, 2019	II	33 pts with MMRd/MMRp recurrent or persistent EC	Avelumab 10 mg/kg IV every 2 weeks	**MMRd cohort** ORR: 26.7% PFS6: 40% *MMRp cohort was closed at the first stage because of futility
Marabelle et al., 2020 (Keynote-158)	II	233 pts with MSI/MMRd advanced non-CRC	Pembrolizumab 200 mg IV every 3 weeks	ORR: 34.3% PFS: 4.1 months OS: 23.5 months
Antill, 2017 (PHAEDRA study)	II	71 pts with advanced or unresectable MMRd/MMRp EC	Durvalumab 1500 mg IV every 28 days until disease progression or prohibitive toxicity	**MMRd** OTR: 40% **MMRp** OTR: 3%
Oaknin, 2019 (GARNET study)	II	110 pts with recurrent or advanced MSI/MSS EC	Dostarlimab 500 mg IV every 3 weeks	**MSI cohort** ORR: 50% **MSS cohort** ORR: 19.1

Abbreviations: ORR, overall response rate; OS, overall survival; PFS, progression-free survival; PFS12, progression-free survival at 12 months; PFS6, progression-free survival at 6 months; OTR, objective tumor response; DOR, duration of response; MMRd, mismatch repair deficient; MMRp, mismatch repair proficient; EC, endometrial cancer; MSI, microsatellite instability; MSS, microsatellite stability; IV, intravenous; pts, patients; CRC, colorectal cancer.

**Table 2:** Ongoing clinical trials on immune checkpoint inhibitors in endometrial cancer.

DRUGS	Description/Condition	Setting	Primary Endpoint	Phase	Status	Trial Identifier
Nivolumab	A Phase Ib/IIa Study of Rucaparib (PARP Inhibitor) Combined with Nivolumab in Metastatic Castrate—Resistant Prostate Cancer and Advanced/Recurrent Endometrial Cancer	Advanced/Recurrent EC	DLT	I/II	Recruiting	NCT03572478
Nivolumab	A Phase II Trial of IDO-Inhibitor, BMS-986205, and PD-1 Inhibitor, Nivolumab, in Patients with Recurrent or Persistent Endometrial Cancer or Endometrial Carcinosarcomas (CA017-056)	Recurrent/Persistent EC	ORR	II	Recruiting	NCT04106414
Nivolumab + Ipilimumab	A Phase II Single Arm Study Assessing Efficacy and Safety of Nivolumab Plus Ipilimumab in Nonresectable/Metastatic Sarcoma and Endometrial Carcinoma Patients with Somatic Deficient MMR as a Selection Tool	EC with Somatic MMRd	ORR	II	Not yet Recruiting	NCT02982486
Nivolumab	Phase II Trial of Single-Agent Nivolumab in Patients with Microsatellite Unstable/Mismatch Repair Deficient/Hypermutated Uterine Cancer	Metastatic/Recurrent EC	PFS	II	Recruiting	NCT03241745
Nivolumab +/− Ipilimumab	Phase Ib Clinical Investigation of Intraperitoneal Ipilimumab and Nivolumab in Patients with Peritoneal Carcinomatosis Due to Gynecologic Cancers	Recurrent EC	MTD	I	Recruiting	NCT03508570
Nivolumab	A Phase 1a/1b Study of COM701 as Monotherapy and in Combination with an Anti-PD-1 Antibody in Subjects with Advanced Solid Tumors	Advanced or Metastatic Solid Tumor included EC	MTD, DLT, AE	I	Recruiting	NCT03667716
Nivolumab	Targeted Therapy Directed by Genetic Testing in Treating Patients with Advanced Refractory Solid Tumors, Lymphomas, or Multiple Myeloma (The MATCH Screening Trial)	Advanced or Metastatic Solid Tumor included EC	ORR	II	Recruiting	NCT02465060
Pembrolizumab	A Phase 2, Two-Stage Study of Mirvetuximab Soravtansine (IMGN853) in Combination with Pembrolizumab in Patients with Microsatellite Stable (MSS) Recurrent or Persistent Endometrial Cancer (EC)	Advanced/Recurrent EC	ORR, PFS	II	Recruiting	NCT03835819
Pembrolizumab	A Phase II Evaluation of Pembrolizumab, a Humanized Antibody Against PD-1, in the Treatment of Persistent or Recurrent Hypermutated/Ultramutated Endometrial Cancer Identified by Next Generation Sequencing (NGS) and Comprehensive Genomic Profiling (CGP)	Persistent/Recurrent EC	ORR, AE	II	Recruiting	NCT02899793
Pembrolizumab	A Phase Ib Trial of Vaginal Cuff Brachytherapy + Pembrolizumab (MK3475) Followed by 3 Cycles of Dose Dense Paclitaxel/q 21 Day Carboplatin + Pembrolizumab (MK3475) in High Intermediate Risk Endometrial Cancer	High/Intermediate Risk EC	Proportion of patients completing three cycles	I	Not yet Recruiting	NCT03932409
Pembrolizumab	Pembrolizumab with Axitinib in Recurrent Endometrial Cancer with Deficient Mismatch Repair System Post PD1 Exposure: Phase II Trial	Recurrent EC with MMRd	ORR	II	Not yet recruiting	NCT04197219
Pembrolizumab	Phase II Study of Pembrolizumab in Combination with Carboplatin and Paclitaxel for Advanced or Recurrent Endometrial Adenocarcinoma	Advanced or Recurrent EC	ORR	II	Recruiting	NCT02549209
Pembrolizumab	Study of Pembrolizumab Combined with Ataluren in Patients with Metastatic pMMR and dMMR Colorectal Adenocarcinomas or Metastatic dMMR Endometrial Carcinoma: the ATAPEMBRO Study	Metastatic EC MMRd	AE	I/II	Recruiting	NCT04014530
Pembrolizumab	Immunotherapy with MK-3475 in Surgically Resectable Endometrial Carcinoma	Advanced EC	AE	I	Active, not recruiting	NCT02630823
Pembrolizumab	Phase II Trial of Pembrolizumab in Combination with Doxorubicin in Advanced, Recurrent or Metastatic Endometrial Cancer (TOPIC)	Advanced or Metastatic EC	PFS	II	Active, not recruiting	NCT03276013
Pembrolizumab	A Multicenter, Open-label, Randomized, Phase 3 Trial to Compare the Efficacy and Safety of Lenvatinib in Combination with Pembrolizumab Versus Treatment of Physician’s Choice in Participants with Advanced Endometrial Cancer	Advanced EC	PFS, OS	III	Active, not recruiting	NCT03517449
Pembrolizumab	A Phase III Randomized, Placebo-Controlled Study of Pembrolizumab (MK-3475, NSC #776864) in Addition to Paclitaxel and Carboplatin for Measurable Stage III or IVA, Stage IVB, or Recurrent Endometrial Cancer	Advanced or Recurrent EC	PFS	III	Recruiting	NCT03914612
Pembrolizumab	A Phase III Randomized Trial of Radiation +/− MK-3475 (Pembrolizumab) for Newly Diagnosed, High Intermediate Risk Mismatch Repair Deficient (dMMR) Endometrioid Endometrial Cancer	Early stage EC	3 years RFS	III	Not yet recruiting	NCT04214067
Pembrolizumab	A Phase 3 Randomized, Open-Label, Study of Pembrolizumab (MK-3475) Plus Lenvatinib (E7080/MK-7902) Versus Chemotherapy for First-Line Treatment of Advanced or Recurrent Endometrial Carcinoma (LEAP-001)	Advanced or Recurrent EC	PFS, OS	III	Recruiting	NCT03884101
Pembrolizumab	A Phase II Investigation of Pembrolizumab (Keytruda) in Combination with Radiation and an Immune Modulatory Cocktail in Patients with Cervical and Uterine Cancer (PRIMMO Trial)	Advanced or Refractory EC	ORR	II	Recruiting	NCT03192059
Pembrolizumab	A Pilot Study Investigating the Effect of Pembrolizumab on the Tumoral Immunoprofile of Gynecologic Cancers of Mullerian Origin	EC any stages	Change in tumor Immune Infiltrates	Early phase I	Active, not recruiting	NCT02728830
Pembrolizumab	A Phase 1a/1b Study of FPA150, an Anti-B7-H4 Antibody, in Patients with Advanced Solid Tumors	Advanced EC	MTD	Early phase I	Active, not recruiting	NCT03514121
Pembrolizumab	A Platform Study Exploring the Safety, Tolerability, Effect on the Tumor Microenvironment, and Efficacy of Pembrolizumab + INCB Combinations in Advanced Solid Tumors	Advanced or Metastatic EC	AE	I	Active, not recruiting	NCT02646748
Pembrolizumab	A Phase 1/1b Multicenter Study to Evaluate the Humanized Anti-cd73 Antibody, cpi-006, as a Single Agent or in Combination with Ciforadenant, with Pembrolizumab, and with Ciforadenant plus Pembrolizumab in Adult Subjects with Advanced Cancers	Advanced EC	DLT, AE, MDL	I	Recruiting	NCT03454451
Pembrolizumab	A Phase 1/2 Study Exploring the Safety, Tolerability, and Efficacy of MK-3475 in Combination with INCB024360 in Subjects with Selected Cancers (ECHO-202/KEYNOTE-037)	Advanced or Metastatic EC	AE, ORR	I/II	Active, not recruiting	NCT02178722
Pembrolizumab	A Clinical Trial of Pembrolizumab (MK-3475) Evaluating Predictive Biomarkers in Subjects with Advanced Solid Tumors (KEYNOTE 158)	Advanced EC	ORR	II	Recruiting	NCT02628067
Pembrolizumab	A Phase 1/2 Safety and Efficacy Study of INCAGN01876 in Combination with Immune Therapies in Subjects with Advanced or Metastatic Malignancies	Advanced or Metastatic EC	AE, ORR, CRR	I/II	Active, not recruiting	NCT03277352
Pembrolizumab	An Open-Label Phase 1b Trial of Lenvatinib Plus Pembrolizumab in Subjects with Selected Solid Tumors	Refractory EC	AE, DLT	I	Active, not recruiting	NCT03006887
Pembrolizumab	A Phase 1A/B Study to Evaluate the Safety and Tolerability of ETC-1922159 in Advanced Solid Tumours	Advanced, Metastatic or Refractory EC	MTD, AE	I	Active, not recruiting	NCT02521844
Avelumab	A Phase 2, Two-Group, Two-Stage, Open-Label Study of Avelumab (MSB0010718C) in Patients with MSS, MSI-H, and POLE-mutated Recurrent or Persistent Endometrial Cancer and of Avelumab (MSB0010718C)/Talazoparib (MDV3800, BMN 673) in Patients with MSS Recurrent or Persistent Endometrial Cancer	Recurrent or Metastatic EC	PFR6	II	Recruiting	NCT02912572
Avelumab	MITO END-3: A Randomized Phase II Trial of Carboplatin + Paclitaxel Compared to Carboplatin + Paclitaxel + Avelumab in Advanced (Stages III–IV) or Recurrent Endometrial Cancer	Advanced or Recurrent EC	PFS	II	Not yet recruiting	NCT03503786
Durvalumab	Durvalumab and Olaparib in Metastatic or Recurrent Endometrial Cancer	Advanced or Metastatic EC	PFS	II	Recruiting	NCT03951415
Durvalumab	A Phase 2 Trial of Durvalumab (MEDI4736) (Anti-PD-L1 Antibody) with or without Tremelimumab (Anti-CTLA-4 Antibody) in Patients with Persistent or Recurrent Endometrial Carcinoma and Endometrial Carcinosarcoma	Recurrent or Persistent EC	ORR	II	Recruiting	NCT03015129
Durvalumab	A Randomised, Multicentre, Double-blind, Placebo-controlled, Phase III Study of First-Line Carboplatin and Paclitaxel in Combination with Durvalumab, Followed by Maintenance Durvalumab with or without Olaparib in Patients with Newly Diagnosed Advanced or Recurrent Endometrial Cancer (DUO-E)	Advanced or Recurrent EC	PFS	III	Not yet recruiting	NCT04269200
Durvalumab	A Phase 1 Study of Durvalumab, Tremelimumab, and Radiotherapy in Recurrent Gynecologic Cancer	Metastatic or Unresectable EC	MTD	I	Recruiting	NCT03277482
Durvalumab	Pilot Study of Durvalumab (MEDI4736) in Combination with Vigil in Advanced Women’s Cancers	Advanced EC	AE	II	Not yet recruiting	NCT02725489
Durvalumab	Phase 1B, Open-Label, Dose Escalation, and Cohort Expansions Trial of Naptumomab Estafenatox (Nap, ABR-217620) in Combination with Durvalumab (MEDI4736) in Subjects with Selected Advanced or Metastatic Solid Tumors	Advanced EC	AE, MTD, RP2D	II	Recruiting	NCT03983954
Atezolizumab	Phase III Double-blind Randomized Placebo Controlled Trial of Atezolizumab in Combination with Paclitaxel and Carboplatin in Women with Advanced/Recurrent Endometrial Cancer	Advanced or Recurrent EC	OS, PFS	III	Recruiting	NCT03603184
Atezolizumab	A Phase II, Single Arm Study of Atezolizumab + Bevacizumab in Women with Advanced, Recurrent, or Persistent Endometrial Cancer	Recurrent EC	OTR	II	Recruiting	NCT03526432
Atezolizumab	An Open Label, Non-Randomized Multisite Phase II Trial Combining Bevacizumab, Atezolizumab, and Rucaparib for the Treatment of Previously Treated Recurrent and Progressive Endometrial Carcinoma	Recurrent or Progressive EC	ORR	II	Recruiting	NCT03694262
Atezolizumab	A Phase 1b to Assess the Safety and Tolerability of Carboplatin-Cyclophosphamide Combined with Atezolizumab, an Antibody that Targets Programmed Death Ligand 1 (PD-L1), in Patients with Advanced Breast Cancer and Gynaecologic Cancer	Advanced EC	Toxicity	I	Active, not recruiting	NCT02914470
Atezolizumab	A Phase 1b Dose-Escalation Study of Cabozantinib (XL184) Administered Alone or in Combination with Atezolizumab to Subjects with Locally Advanced or Metastatic Solid Tumors	Advanced or Metastatic EC	MTD, ORR	I/II	Recruiting	NCT03170960

Abbreviations: ORR, overall response rate; OS, overall survival; PFS, progression-free survival; PFS6 progression-free survival at 6 months; DLT, dose-limiting toxicities; MTD, maximum tolerated dose; OTR, objective tumor response; DOR, duration of response; RP2D, recommended phase 2 dose; AE, adverse event; MMRd, mismatch repair deficient; MMRp, mismatch repair proficient; EC, endometrial cancer.
